# Cost-Effectiveness Analysis of Pembrolizumab Plus Chemotherapy vs. Chemotherapy Alone as First-Line Treatment in Patients With Esophageal Squamous Cell Carcinoma and PD-L1 CPS of 10 or More

**DOI:** 10.3389/fpubh.2022.893387

**Published:** 2022-06-14

**Authors:** Zhiwei Zheng, Jingrong Lin, Huide Zhu, Hongfu Cai

**Affiliations:** ^1^Department of Pharmacy, Cancer Hospital of Shantou University Medical College, Shantou, China; ^2^Department of Pharmacy, Fujian Medical University Union Hospital, Fuzhou, China

**Keywords:** cost-effectiveness, KEYNOTE-590 clinical, esophageal squamous cell carcinoma, chemotherapy, pembrolizumab

## Abstract

**Background:**

This study aimed to analyze the economics of pembrolizumab plus chemotherapy as first-line treatment in patients with esophageal squamous cell carcinoma (ESCC) and programmed cell death-Ligand 1 (PD-L1) combined positive score (CPS) of 10 or more in China.

**Methods:**

Based on the advanced ESCC of the KEYNOTE-590 clinical trial data, a Markov model was performed to simulate the clinical course and evaluate the patient's total lifetime, total costs, quality-adjusted life-years (QALYs), and incremental cost-effectiveness ratio (ICER) for pembrolizumab plus chemotherapy (cisplatin and 5-fluorouracil) vs. chemotherapy alone in first-line treatment of ESCC and PD-L1 CPS of 10 or more. Utility values and direct costs related to the treatments were gathered from the published literature data. One-way and probabilistic sensitivity analyses were conducted to check the stability of the model.

**Results:**

The baseline analysis indicated that the incremental effectiveness and cost of pembrolizumab plus chemotherapy vs. chemotherapy alone added 1.23 QALYs and resulted in an incremental cost of $51,320.22, which had an ICER of $41,805.12/QALY, higher than the willingness-to-pay (WTP) threshold of China ($37,663.26/QALY). The sensitivity analysis demonstrated that the ICERs were most sensitive to the cycle of pembrolizumab used and the cost of pembrolizumab.

**Conclusion:**

The result of our present analysis suggests that the addition of pembrolizumab plus chemotherapy as first-line treatment might not be cost-effective for patients with ESCC and PD-L1 CPS of 10 or more in China.

## Introduction

Esophageal cancer (EC) is one of the most common malignant tumors in the world. The crude mortality rate of EC was 7.8/100,000 in 2020, which represented 5.5% of all cancer deaths and ranked as the sixth most common cause of cancer death (1). In China, EC is the fourth most common cause of mortality, with 30.1 deaths per 100,000 in 2020 (2, 3). Esophageal squamous cell carcinoma (ESCC) is the major histological type of esophageal cancer, which accounts for about 90% of the 456,000 incident esophageal cancers each year (4). Despite advances in the multidisciplinary treatment of ESCC, treatment options for unresectable, locally advanced, or metastatic esophageal cancer are still limited (5). For patients with advanced or metastatic ESCC, a combination of 5-fluoropyrimidine and platinum-based chemotherapy was recommended as first-line therapy (6, 7). However, the overall 5 years survival rate of ESCC was poor and reported as 20.9% in China (8). Therefore, the treatment of ESCC has gradually become a more and more difficult problem and new treatment strategies are urgently needed.

In recent years, immunotherapy and immune checkpoint inhibitors (ICIs) have provided new therapeutic options and shown good performance in ESCC (9, 10). Among them, pembrolizumab used alone provided an overall response rate of 14% in ESCC and PD-L1 combined positive score (CPS) of 10 or more as third-line therapy in phase 2 KEYNOTE-180 study (11). Another phase III KEYNOTE-181 study showed that pembrolizumab provided a median overall survival of 10.3 vs. 6.7 months with chemotherapy as second-line therapy in patients with ESCC and PD-L1 CPS of 10 or more (12).

Based on phase III clinical trial KEYNOTE-590, the National Medical Products Administration of China approved pembrolizumab for treating patients with advanced or metastatic ESCC whose tumors express PD-L1 CPS of 10 or more (13). The KEYNOTE-590 trial was conducted to evaluate the efficacy of pembrolizumab plus chemotherapy vs. chemotherapy alone for first-line treatment of advanced ESCC. The result showed that pembrolizumab plus chemotherapy significantly prolonged median progression-free survival (PFS median 7.5 vs. 5.5 months) and median overall survival (OS median 13.9 vs. 8.8 months) compared with chemotherapy.

The outstanding performance with significant improvements in PFS and OS showed the apparent benefit of pembrolizumab treatment as first-line with advanced ESCC. However, the high cost of pembrolizumab could have far-reaching economic consequences. Hence, the purpose of our study was to analyze the economics of pembrolizumab plus chemotherapy for first-line treatment in patients with ESCC and PD-L1 CPS of 10 or more based on the KEYNOTE-590 trial from the perspective of the Chinese healthcare system.

## Methods

### Model Structure

**Figure 1:**
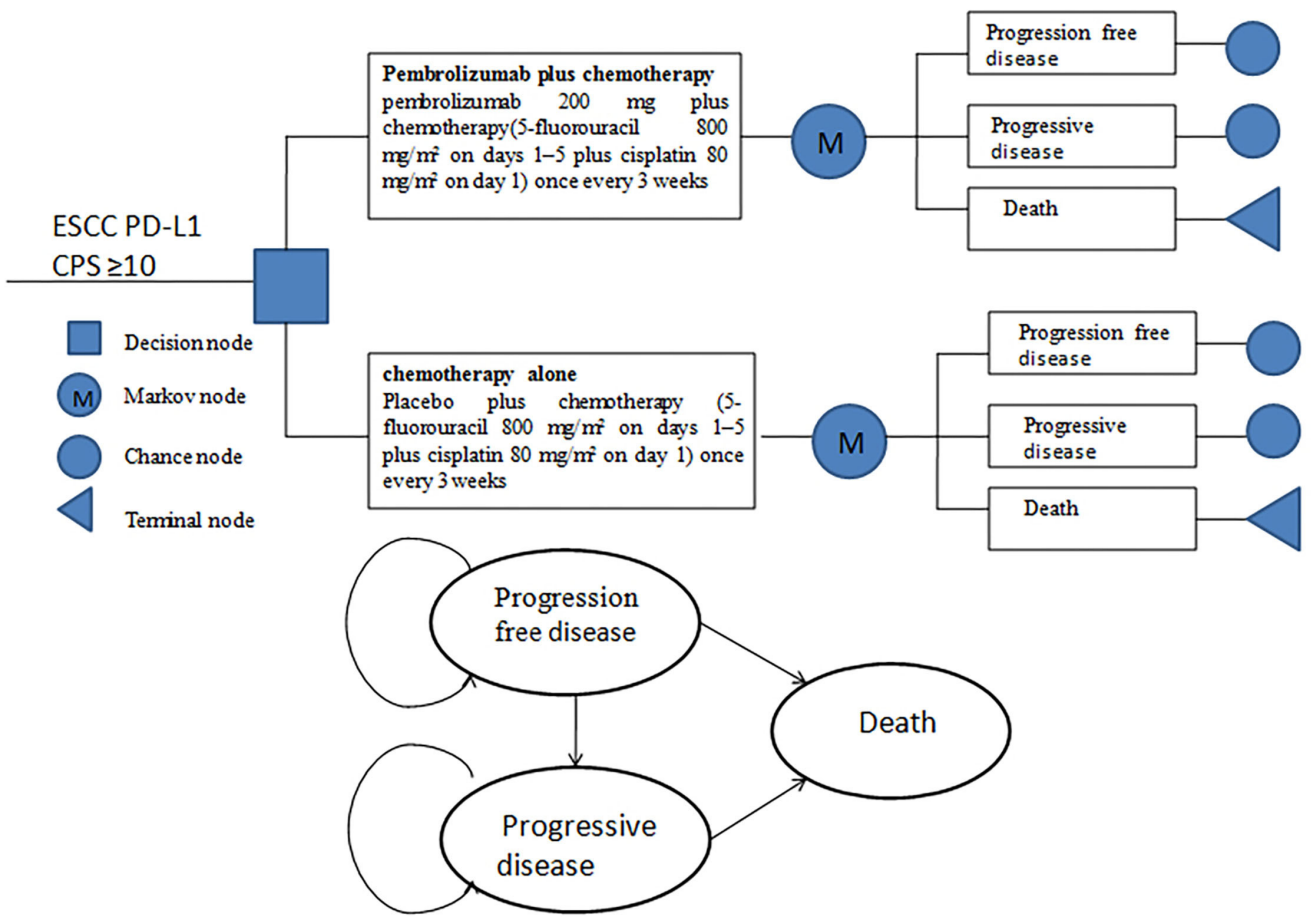
Model structure simulated three health states: progression-free disease, progressive disease, and death.

A Markov model was established to analyze the clinical and economic outcomes of pembrolizumab plus chemotherapy vs. chemotherapy alone as first-line therapy for patients with advanced or metastatic ESCC and PD-L1 CPS of 10 or more in China. The model included three mutually exclusive health states: progression-free disease (PFD), progressive disease (PD), and death (Figure 1). Due to the overall 5-year survival rate for people with ESCC, being ~20% or less (8), the time horizon of the model was set to 10 years. As patients received pembrolizumab or chemotherapy once every 3 weeks in the KEYNOTE-590 trial, the model period was set to 21 days. The primary outcomes were total life years, total cost, ICER, and QALYs in the study. ICER refers to the additional cost required for each additional QALY. The future costs and benefits were discounted at a rate of 5% according to the practice of pharmacoeconomic evaluation guidelines for universal health coverage in China (14). All costs were presented in US dollars, with an average RMB exchange rate of $1 to 6.45 Yuan for the full year of 2021. In addition, 3 × the per capita gross domestic product (GDP) of China in 2021 ($37,663.26) was used as the willingness-to-pay (WTP) threshold according to recommendations (15, 16). The TreeAge Pro 2011 software package (Williamstown, MA, USA) and R software (version 4.0.5, Vienna, Austria) were used to build the model and conduct statistical analysis.

### Clinical Data

The clinical efficacy and safety data were based on the patients in the KEYNOTE-590 trial, a randomized, placebo-controlled, and multicenter phase 3 study that enrolled patients from 168 medical centers in 26 countries. Patients aged 18 years or older with previously untreated, histologically or cytologically confirmed, locally advanced, unresectable, or metastatic ESCC were randomly assigned (1:1) to intravenous pembrolizumab 200 mg or placebo, plus 5-fluorouracil, and cisplatin (chemotherapy), once every 3 weeks. The duration of treatment exposure was 7.7 ± 6.84 months in the pembrolizumab plus chemotherapy group and 5.8 ± 4.76 months in the placebo plus chemotherapy group.

Pembrolizumab plus chemotherapy outperformed chemotherapy in terms of overall survival [median 13.9 vs. 8.8 months; hazard ratio 0.57 (95% CI 0.43–0.75); *p* < 0.0001] and median progression-free survival [7.5 vs. 5.5 months; 0.51 (0.41–0.65); *p* < 0.0001].

**Figure 2:**
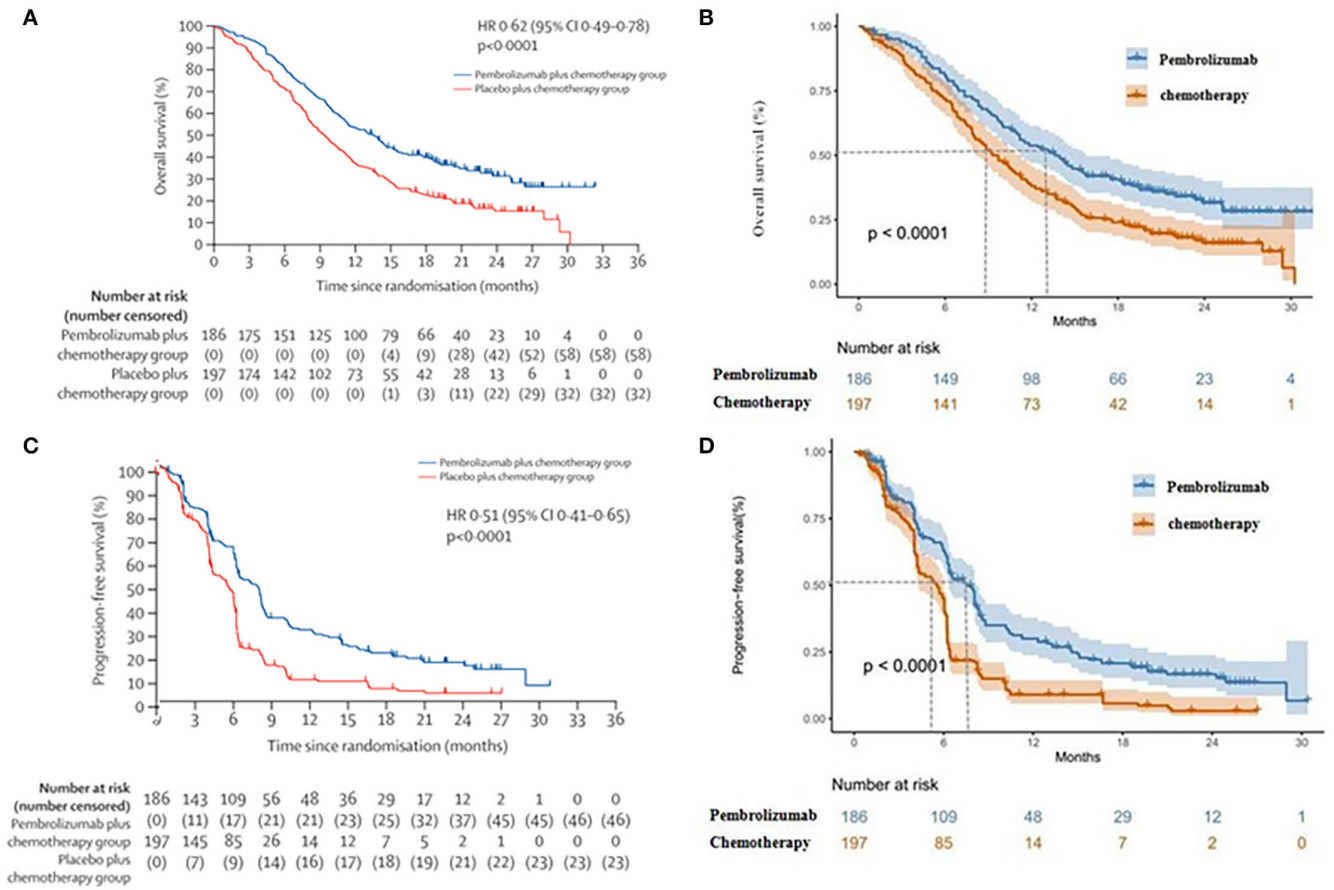
**(A)** Kaplan–Meier curve of the overall survival from the KEYNOTE-590 trial. **(B)** Simulate overall survival curve for pembrolizumab group and chemotherapy group. **(C)** Kaplan–Meier curve of progression-free survival from the KEYNOTE-590 trial. **(D)** Simulate progression-free survival curve for pembrolizumab group and chemotherapy group.

The grade 3–4 adverse events were selected from the KEYNOTE-590 trial based on two principles: (1) Any adverse events occurred in both the pabolizumab and placebo groups >30%; (2) The difference in grade 3–4 adverse events between the two groups was >3%. The probability of transition between different health states was evaluated from the Kaplan–Meier survival curve, which was obtained from the KEYNOTE-590 trial. The Get Data Graph Digitizer 2.25 (http://www.getdata-graph-digitizer.com) was used to read points on the Kaplan–Meier curves of PFS and OS for the two groups. To extrapolate the probability of survival, R software was used to remodel the individual data, which were then simulated by Weibull, Log-logistic, Log-normal, Gompertz, Exponential, and Gamma. The best suitable distribution was selected both by visual inspection and remodeling data, which was the minimum value of the Akaike information criterion (AIC) and the Bayesian information criterion (BIC). The log-logistic distribution function was finally selected to simulate the PFS and OS curves of the two schemes. The survival curve simulation results are present in Figure 2. The internal validation demonstrated that the PFS and OS curves closely approximated those presented in the clinical trials (Supplementary Figures S1–S4). The estimated scale (λ), shape (γ), and key clinical parameters were presented in Table 1. The *S*(*t*) =1/(1 + λ*t*^γ^) with the scale (λ) parameter and the shape (γ) parameter was used to calculate the survival function of a log-logistic distribution over time (22).

**Table 1:** Model economic parameters and the range of the sensitivity analysis.

**Variable**	**Baseline value**	**Range**		**Distribution**	**Source**
		**Minimum**	**Maximum**		
**Log-logistic OS survival model**
Pembrolizumab group	shape (γ) = 1.59; scale (λ) = 0.015	–	–	–	(13)
Chemotherapy group	shape (γ) = 1.70; scale (λ) = 0.022	–	–	–	(13)
**Log-logistic PFS survival model**
Pembrolizumab group	shape (γ) = 1.75; scale (λ) = 0.029	–	–	–	(13)
chemotherapy group	shape (γ) = 2.27; scale (λ) = 0.028	–	–	–	(13)
**Drug cost per mg, US $**
pembrolizumab per mg	25.98	12.99	25.98	Gamma	(17)
5-fluorouracil per mg	0.03956	0.03297	0.04239	Gamma	Local charge
cisplatin per mg	0.1036	0.1036	0.1463	Gamma	Local charge
docetaxel per mg	1.77	0.26	14.95	Gamma	(18)
rinotecan per mg	1.64	0.88	4.65	Gamma	(18)
**Costs of serious adverse events ($)**
Anemia	73.68	55.27	92.11	Gamma	(19)
Decreased neutrophil count	67.56	55.27	200.66	Gamma	(19)
**Pembrolizumab group AEs (grade** **≥3) incidence (%)**
Anemia	12	9.6	1.44	Beta	(13)
Decreased neutrophil count	23	18.4	27.6	Beta	(13)
**Chemotherapy group AEs (grade** **≥3) incidence (%)**
Anemia	15	12	18	Beta	(13)
Decreased neutrophil count	17	13.6	20.4	Beta	(13)
**Utility value**
Progression-free disease	0.741	0.593	0.889	Beta	(18)
Progressive disease	0.581	0.465	0.697	Beta	(18)
Anemia	−0.074	−0.110	−0.037	Beta	(18)
Decreased neutrophil count	−0.090	−0.120	−0.059	Beta	(18)
Follow-up cost per cycle	51.5	45	58.4	Beta	(20)
Body surface area, m 2	1.72	1.5	1.9	Beta	(14, 19)
Discount rate	0.05	0	0.08	Beta	(21)

### Costs and Utilities

The costs were estimated from the Chinese perspective. The model included only direct medical expenses, such as the cost of pembrolizumab and chemotherapy, treatment-related grade 3–4 serious adverse events (SAEs) management, the cost per cycle of salvage treatment, and routine follow-up (Table 1). By KEYNOTE-590 trial research, patients received pembrolizumab 200 mg or chemotherapy (5-fluorouracil 800 mg/m^2^ on days 1–5 plus cisplatin 80 mg/m^2^ on day 1 [for a maximum of six cycles]) once every 3 weeks for up to 35 cycles. After the failure of first-line treatment, the subsequent therapy of patients receiving second-line chemotherapy regimens was not defined and listed in the clinical trials. Thus, our model assumed that the patients had an equal opportunity to receive docetaxel (75 mg/m^2^, once every 3 weeks) or irinotecan (180 mg/m^2^, once every 2 weeks) for currently recommended second-line chemotherapy (10). We will perform a sensitivity analysis of the second-line chemotherapy opportunity to evaluate the sensitivity impact on economic outcomes. To estimate the dosage of chemotherapeutic drugs, we assume that the typical patient weighed 65 kg and is 160-cm tall, so the body surface area (BSA) is 1.72 m^2^. All costs were calculated using either local charges or previously published literature (17, 23). As no data on quality of life was estimated in the KEYNOTE-590 trial, the utility values for the PFD and PD health states were taken from the literature (18). Death had a zero-utility value, and the model also calculated the disutility produced by SAEs. All utility values are presented in Table 1.

### Sensitivity Analysis

One-way and probabilistic sensitivity analyses (PSA) were conducted to check the stability of the model.

One-way analyses were conducted to check the influence of different parameters on ICER when changed with a range of ±20% of the base case value, to identify the most significantly influenced parameters. The current price of pembrolizumab fluctuated by 50%, as the value range and the discount rate were 0%−8%. The results of the one-way sensitivity analysis were presented in the form of a tornado diagram.

The PSA was conducted to evaluate the overall uncertainty of the research results. A 10,000 Monte Carlo simulation was used to run the model, in which the parameters were set with a specific distribution (gamma distribution for costs, beta distribution for the probability parameters, and utilities). The results of the PSA were presented as scatter plots and cost-effectiveness acceptance curves.

## Results

### Base Case Analysis

The base case analysis showed that over a 10-year time horizon, the pembrolizumab plus chemotherapy group gained total life of 3.87 years and 2.38 QALYs for $61,051.30, while the chemotherapy group gained total life of 1.87 years and 1.16 QALYs for $9,731.08. Compared with chemotherapy, the pembrolizumab plus chemotherapy group showed an incremental cost of $51,320.22; the incremental effectiveness was 1.23 QALYs and the ICER was $41,805.12/QALY (Table 2). Pembrolizumab plus chemotherapy was not cost-effective when compared with chemotherapy alone at the Chinese WTP threshold of $37,663.26/QALY.

**Table 2:** The cost and outcome results of the cost-effectiveness analysis.

**Treatment**	**Total cost ($)**	**Total life years**	**Total QALYs**	**Incremental cost ($)**	**Incremental QALY**	**ICER ($/QALY)**
Pembrolizumab plus chemotherapy	61,051.30	3.87	2.38	51,320.22	1.23	41,805.12
Chemotherapy	9,731.08	1.87	1.16			

*ICER, Incremental cost–effectiveness ratio; QALY, quality-adjusted life year*.

### Sensitivity Analyses

**Figure 3:**
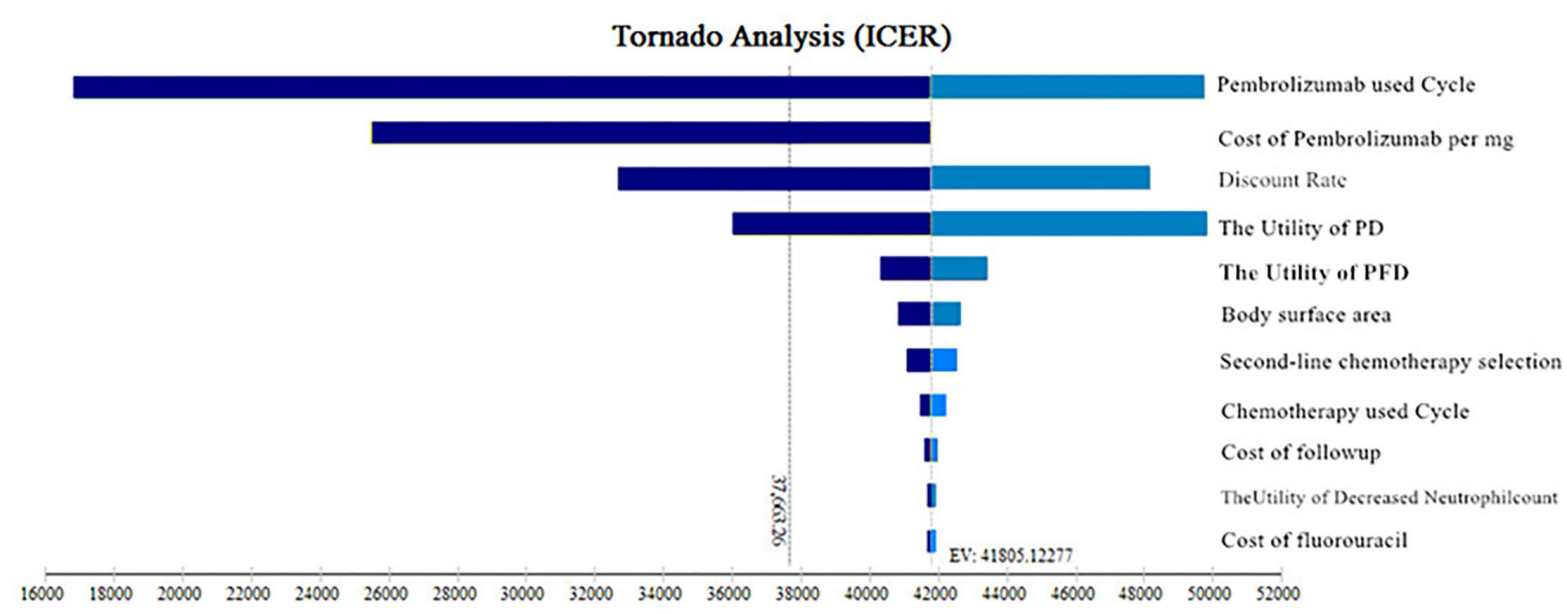
Tornado diagrams of one-way sensitivity analyses. ICER, incremental cost-effectiveness ratios; PFD, progression-free disease; PD, progressive disease.

The one-way sensitivity analysis' tornado diagram was shown in Figure 3. The most influential parameters were the cycle used of pembrolizumab, cost of pembrolizumab, discount rate, and the utility value of PD, which will reduce the ICERs below the thresholds. Other parameters influencing the model were the utility value of PFD, body surface area, second-line chemotherapy selection opportunities, the cycle used in chemotherapy, cost of follow-up, the utility value of decreased neutrophil count, and cost of 5-fluorouracil; whereas none of those variables could reduce the ICERs below the thresholds. Those variables did not change the results.

**Figure 4:**
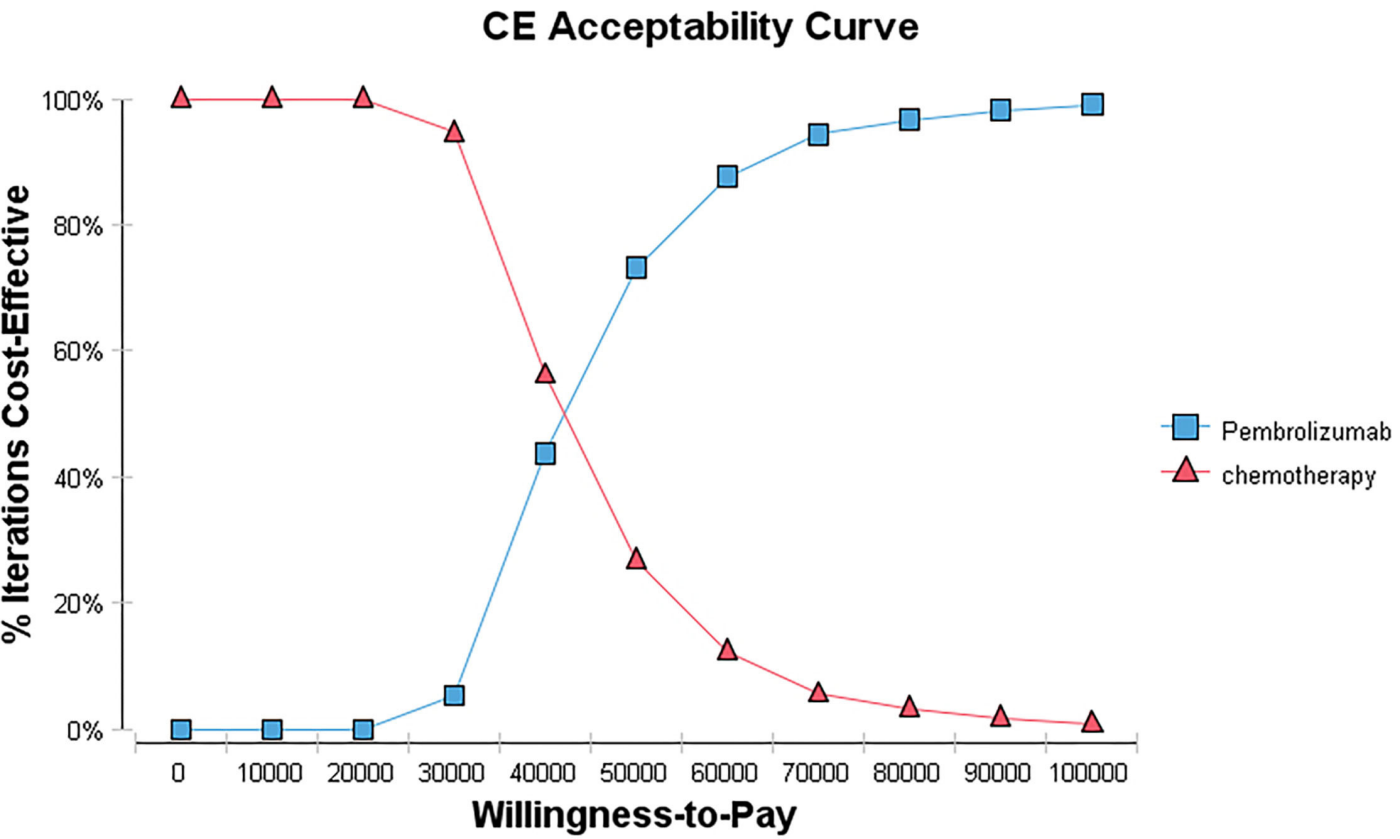
Cost-effectiveness acceptability curve. CE, cost-effectiveness.

**Figure 5:**
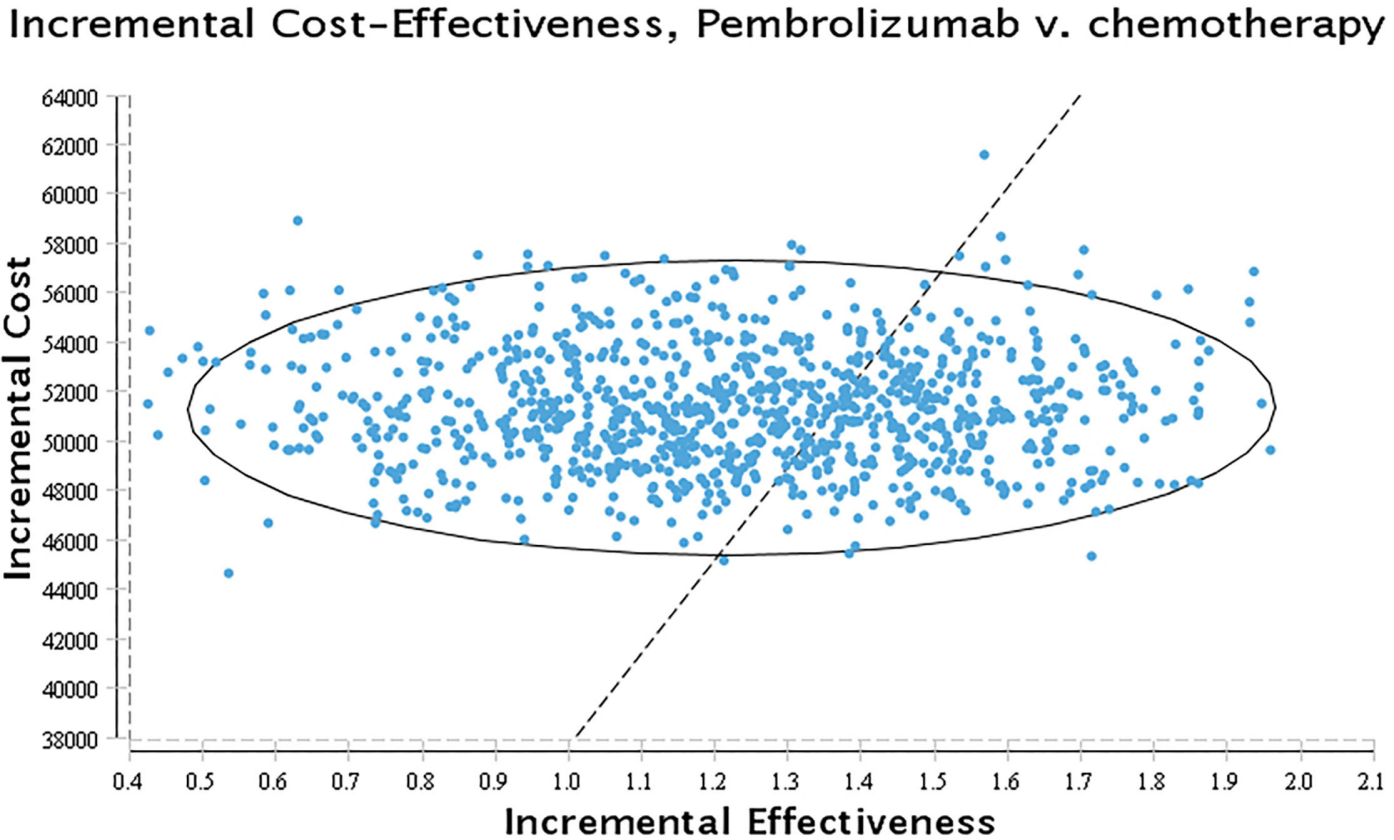
Scatter plot. A probabilistic scatter plot of the ICER between the pembrolizumab and chemotherapy group. Each dot represents the ICER for 1 simulation.

The results of probabilistic sensitivity analysis were presented as a cost-effectiveness acceptability curve and probabilistic scatter plot in Figures 4, 5. The cost-effectiveness acceptability curves represent the results of probabilistic sensitivity analysis by evaluating the chances of different treatments being regarded as optimum strategies at different WTP thresholds. The probabilistic scatter represents the Monte Carlo simulation result, while the ellipse indicates the 95 percent confidence interval (CI). The WTP value is represented by the diagonal line, and the dot false below the diagonal line indicates that the sample population is cost-effective as compared to the control group.

## Discussion

The ESCC is one of the most common malignant tumors globally. Despite advances in the multidisciplinary treatment of ESCC, treatment options for advanced ESCC are limited and had a poor prognosis. In recent years, ICIs have provided a new therapeutic option and have shown good performance in ESCC (24). The results of the KEYNOTE-590 trial show that compared to chemotherapy, pembrolizumab plus chemotherapy significantly prolonged median OS and median PFS in ESCC. It has achieved the longest OS (13.9 months) and the highest response rate (45%) in the field of first-line treatment for ESCC, which provides a new optional first-line treatment for patients with ESCC.

However, the cost of ICIs was high, which could significantly increase expenditures on patients. The high cost of ICIs treatment undoubtedly brings a heavy financial burden to patients. Hence, it is necessary to evaluate the effect of ICIs from the perspective of pharmacoeconomics. For the phase III trial, KEYNOTE-590 was the best choice for cost-effectiveness analysis.

In our present study, the results showed that the ICER of pembrolizumab plus chemotherapy for first-line treatment of advanced ESCC in China was $41,805.12/QALY and the WTP threshold was $37,663.26/QALY, revealing that the pembrolizumab plus chemotherapy strategy was not a cost-effective treatment strategy compared with chemotherapy alone.

The best ICI therapy cycle is presently unknown. The effect of drug treatment duration on the outcomes was taken into account in the constructed model. In the one-way sensitivity analysis, the cycle used for pembrolizumab and the cost of pembrolizumab had the highest impacts on the ICER. However, this variable could not reduce the ICERs below the thresholds. The ICER ($36,449.40/QALY) approached the WTP ($37,663.26/QALY) threshold with cost-effectiveness when the price of pembrolizumab was reduced to $23.90 per mg in China. The greatest impact of the cycle used of pembrolizumab on the ICER was related to the cost of pembrolizumab. Because the current price of pembrolizumab in China was $25.98 per mg, the ICER ($37,083.81/QALY) approached the WTP ($37,663.26/QALY) with cost-effectiveness when the cycle used ≤8 cycles of pembrolizumab. When the price of pembrolizumab was reduced to $23.90 per mg in China, the ICER ($36,449.40/QALY) approached the WTP ($37,663.26/QALY) threshold with cost-effectiveness when the cycle used ≤9 cycles of pembrolizumab. However, there were different cost-effectiveness WTP threshold values in different regions and countries. The ICER of the pembrolizumab plus chemotherapy group was higher than the threshold recommended by the developed countries, such as $15,000 per QALY proposed by the United States (25). WTP is an important criterion for determining if an intervention is cost-effective. If the ICER is less than the WTP, the intervention is considered cost-effective. According to WHO standards that were extensively followed in the last decade, the WTP was set at 1–3 times GDP per capita.

Further, we also specifically modeled the future treatment rather than merely allocating docetaxel as the second-line chemotherapy for all groups. Although we assumed that the patients had an equal opportunity to receive docetaxel or irinotecan for second-line chemotherapy, the sensitivity analysis result showed that the opportunity of selection of second-line chemotherapy could not reduce the ICERs below the thresholds. It did not change the results.

Inhibitors of PD-1 and PD-L1 have become an effective treatment strategy for advanced esophageal cancer (26). Nivolumab, camrelizumab, and pembrolizumab have improved survival and reduced adverse effects in patients with metastatic esophageal cancer. Yang et al. (27) evaluated the cost-effectiveness of camrelizumab vs. chemotherapy as a second-line treatment for patients with advanced ESCC from the perspective of the Chinese. The result showed that camrelizumab was not cost-effective as second-line therapy for advanced or metastatic esophageal squamous cell carcinoma compared with chemotherapy in China. Currently, economic research on pembrolizumab for cancer treatment is very limited, with the majority of studies focusing on colorectal cancer or cervical cancer (28, 29). Zhan et al. (30) published a cost-effectiveness analysis of second-line pembrolizumab treatment in patients with advanced esophageal cancer based on the KEYNOTE-181 study. From the perspective of Chinese society, pembrolizumab is not a cost-effective treatment option for the second-line treatment of esophageal cancer. Pembrolizumab improved survival in patients with a variety of solid tumors, although its economics is debatable due to its high price. The outcomes of the pharmacoeconomic evaluation were influenced by factors such as clinical efficacy, safety, and drug price. When pembrolizumab was treated for patients with advanced ESCC and PD-L1 CPS of 10, our result showed that the price had the greatest influence on the ICER. When the price of pembrolizumab was reduced to $23.90 per mg, the ICER ($36,449.40/QALY) approached the WTP ($37,663.26/QALY) threshold with cost-effectiveness in China.

China has begun nationwide measures to coordinate drug procurement to reduce drug costs. Several Chinese domestic PD-1 inhibitors, including camrelizumab and sintilimab injectable, were included in medical insurance with a >60% price reduction. More PD-1 inhibitors will emerge as the pharmaceutical industry develops, perhaps providing an alternative for patients with esophageal squamous cell carcinoma in China and lowering the price of pembrolizumab. The cost-effectiveness of PD-1 inhibitors will be improved with the price adjustment.

However, our study still has certain limitations. First, the key clinical data in the study was extracted from the clinical trials, which may lead to some bias. Second, we assumed patients had equal opportunities to receive docetaxel or irinotecan for second-line chemotherapy, which may not accurately reflect the real-world condition. However, the result of the sensitivity analysis supported that the second-line chemotherapy selection opportunity did not have an important impact on economic outcomes. Third, only grade 3/4 SAEs were considered in the model. We assumed that grade 1/2 SAEs would not change the result of the study, and then sensitivity analysis demonstrated that the result was not sensitive to SAEs-related parameters. Finally, PFS and PS status utility values were not generated from patients with ESCC. The utility values of PD and PFD were found to be the impacting factors in a one-way sensitivity analysis. The utility values of various treatment schemes for patients with ESCC have not been recovered from published literature, and the only measurement of the utility value of ESCC is the patients with second-line treatment. Despite these limitations, our study may be a valuable reference for doctors and decision-makers about pembrolizumab as a first-line treatment for metastatic ESCC in China.

## Conclusion

Compared with chemotherapy, pembrolizumab plus chemotherapy is unlikely to be considered cost-effective as a first-line treatment for advanced ESCC and PD-L1 CPS of 10 in China. However, pembrolizumab may be a cost-effective treatment option if the price is reduced.

## Data Availability Statement

The original contributions presented in the study are included in the article/Supplementary Material, further inquiries can be directed to the corresponding authors.

## Ethics Statement

Our cost-effectiveness analysis was based on published literature and computer modeling techniques, the study did not require approval from a hospital research ethics board.

## Author Contributions

ZZ and HC designed the study. ZZ written and reviewed the paper. HC and JL supervised the project and assisted with the statistical analysis. HZ performed software analysis. All authors contributed to the article and approved the submitted version.

## Funding

This work was supported by the Science and Technology Special Fund of Guangdong Province of China (190829105556145).

## Conflict of Interest

The authors declare that the research was conducted in the absence of any commercial or financial relationships that could be construed as a potential conflict of interest.

## Publisher's Note

All claims expressed in this article are solely those of the authors and do not necessarily represent those of their affiliated organizations, or those of the publisher, the editors and the reviewers. Any product that may be evaluated in this article, or claim that may be made by its manufacturer, is not guaranteed or endorsed by the publisher.
