# Supplementary figures and images for: Cost-Effectiveness Analysis of Pembrolizumab Plus Chemotherapy vs. Chemotherapy Alone as First-Line Treatment in Patients With Esophageal Squamous Cell Carcinoma and PD-L1 CPS of 10 or More
Source: Front Public Health. 2022 Jun 14;10:893387. doi: 10.3389/fpubh.2022.893387 (PMC9237361; doi:10.3389/fpubh.2022.893387)

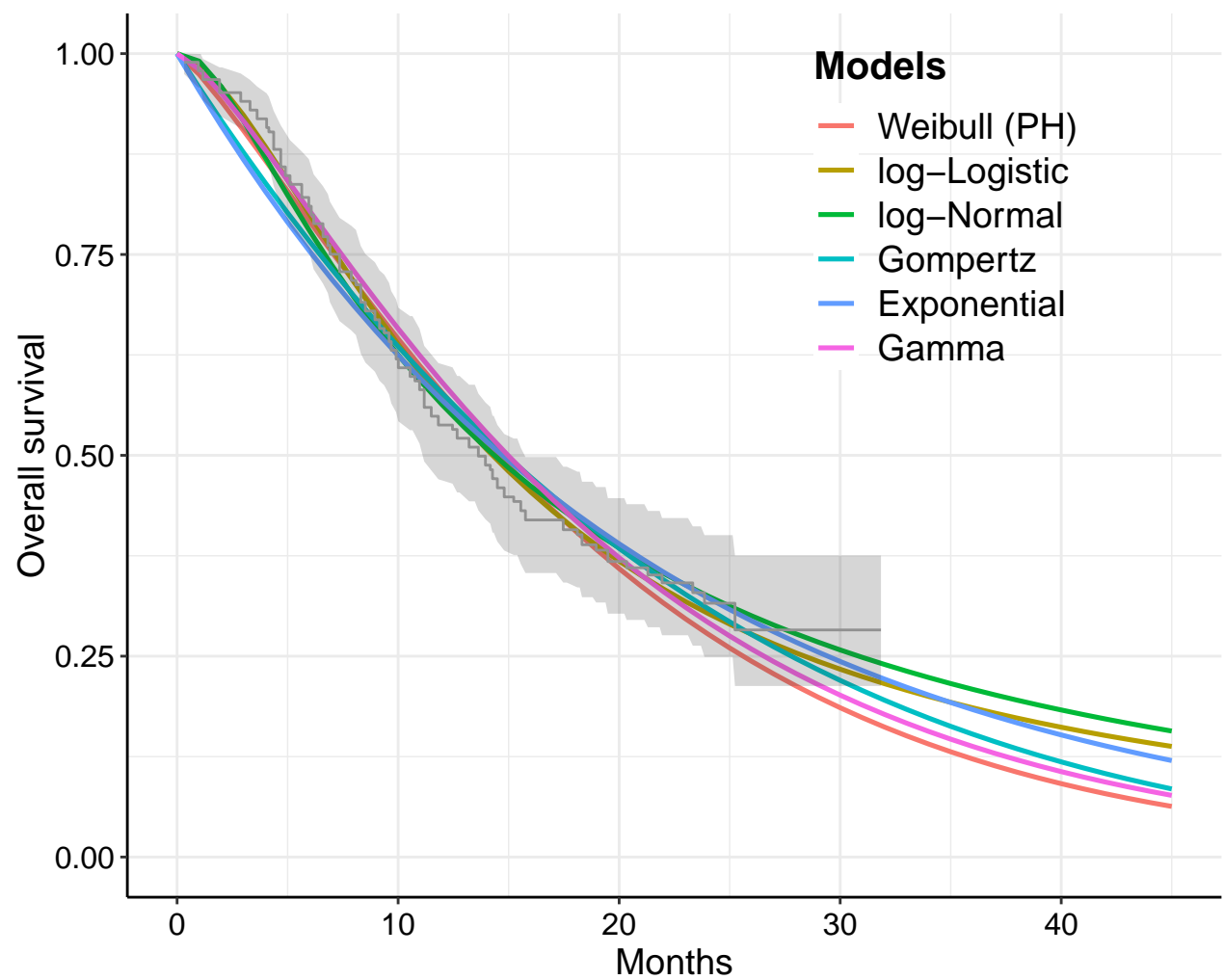

Supplement: Supplementary file 3 [file Data_Sheet_1.PDF]

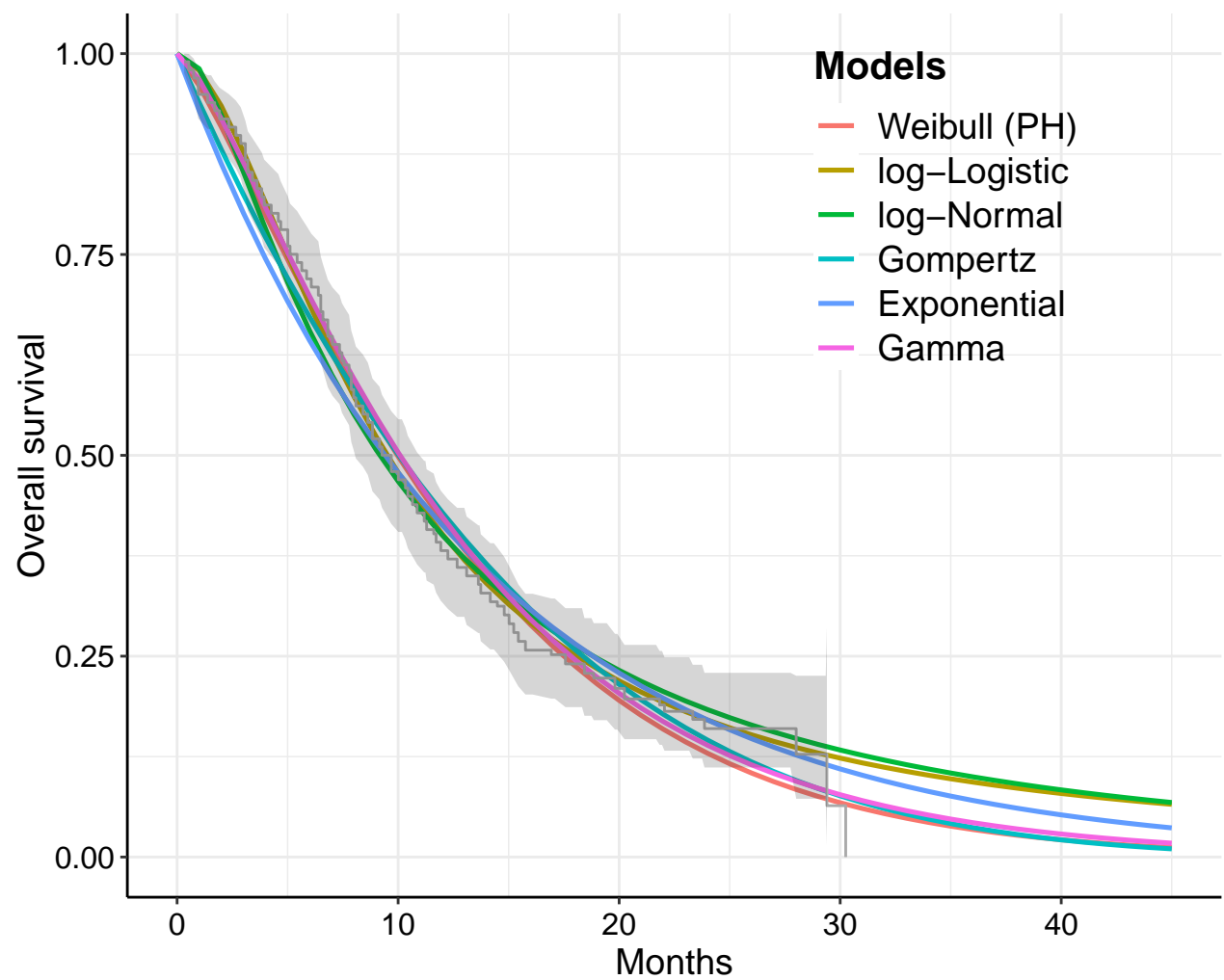

Supplement: Supplementary file 4 [file Data_Sheet_2.PDF]

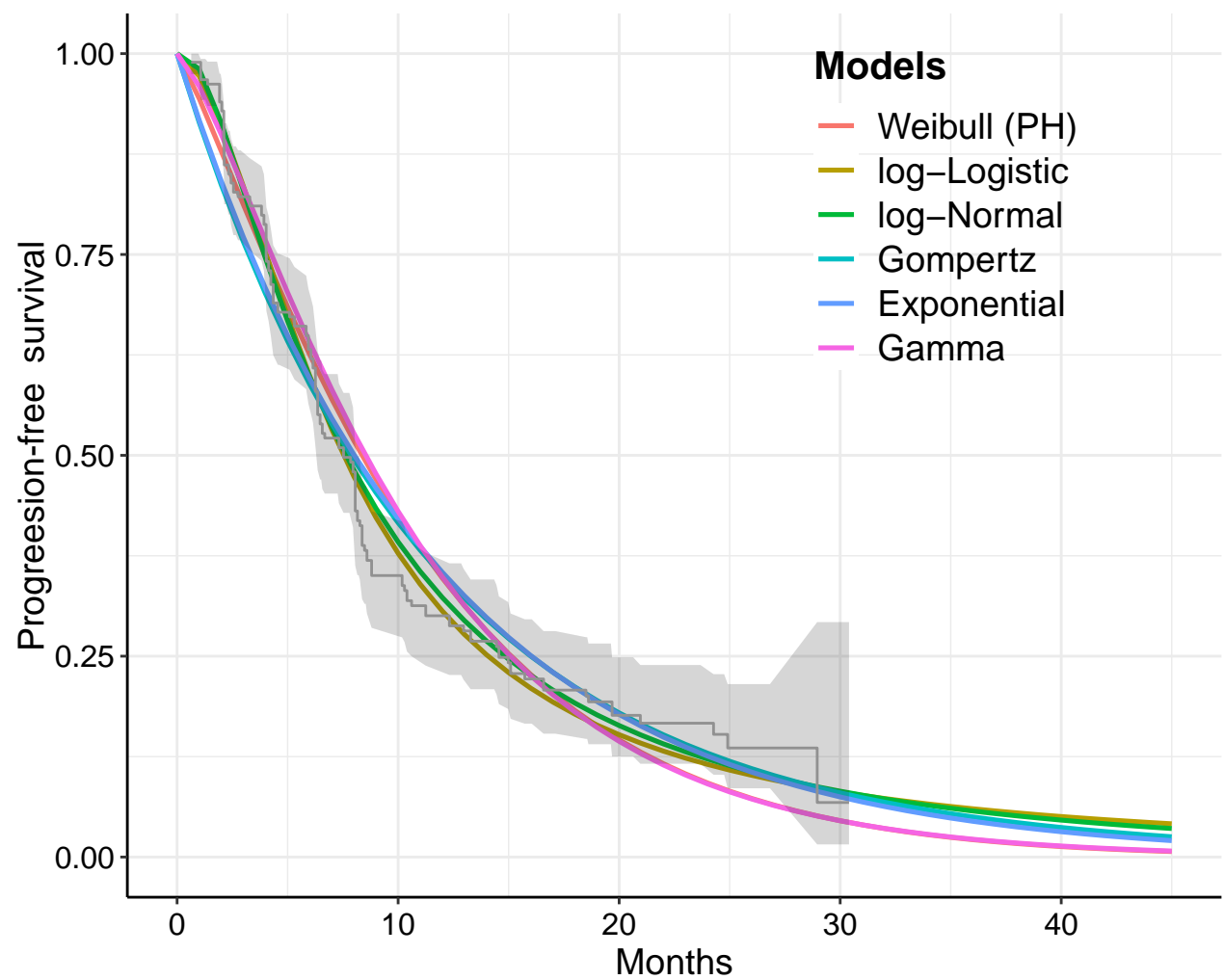

Supplement: Supplementary file 5 [file Data_Sheet_3.PDF]

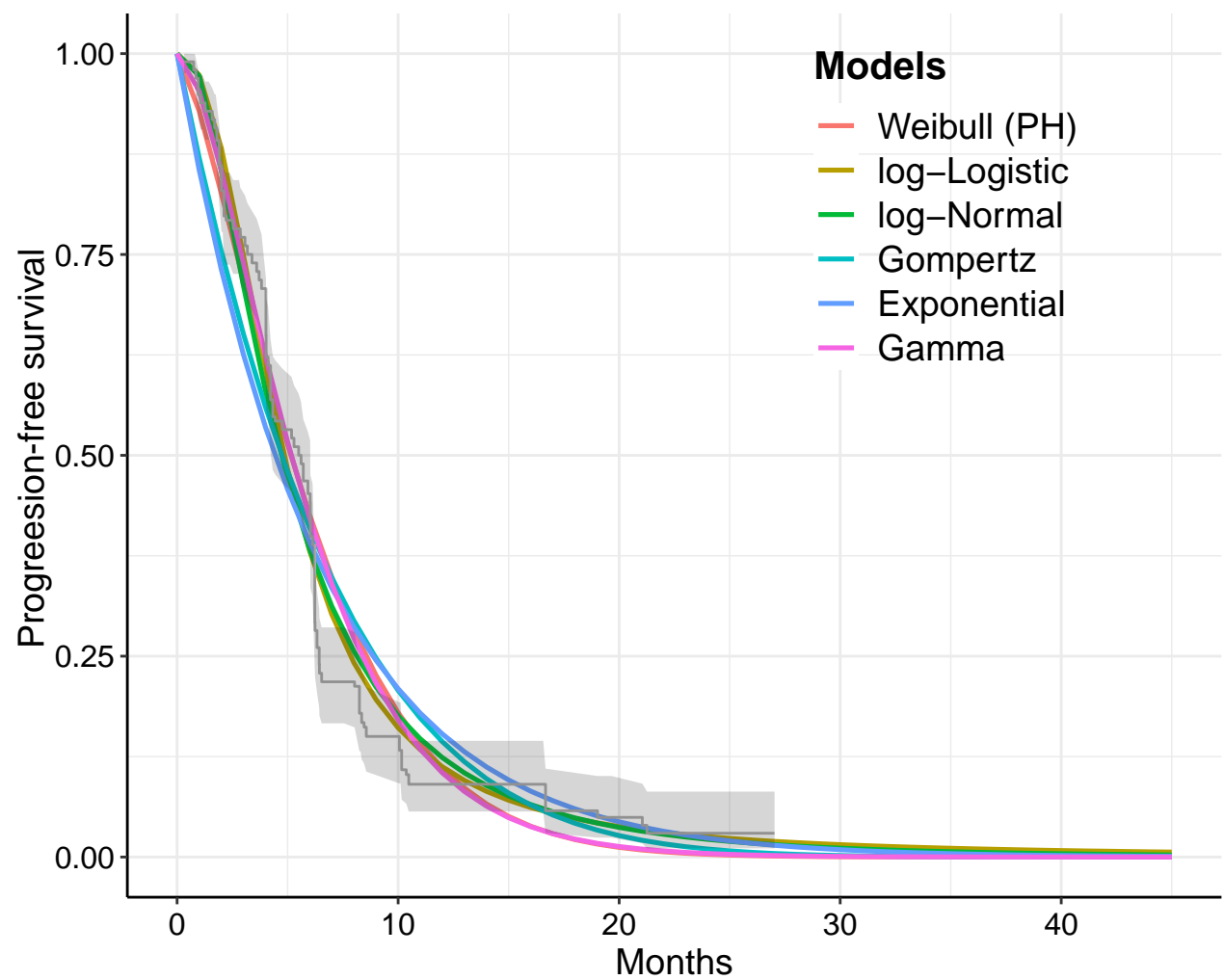

Supplement: Supplementary file 6 [file Data_Sheet_4.PDF]
